# Electrophysiological and histological characterization of atrial scarring in a model of isolated atrial myocardial infarction

**DOI:** 10.3389/fphys.2022.1104327

**Published:** 2023-01-12

**Authors:** Gerard Amorós-Figueras, Sergi Casabella-Ramon, Georgina Company-Se, Dabit Arzamendi, Esther Jorge, Alvaro Garcia-Osuna, Yolanda Macías, Damián Sánchez-Quintana, Javier Rosell-Ferrer, José M. Guerra, Juan Cinca

**Affiliations:** ^1^ Department of Cardiology, Hospital de la Santa Creu i Sant Pau, IIB-Sant Pau, UAB, CIBERCV, Barcelona, Spain; ^2^ Electronic and Biomedical Instrumentation Group, Department of Electronics Engineering, Universitat Politècnica de Catalunya, Barcelona, Spain; ^3^ Biochemistry Department, Hospital de la Santa Creu i Sant Pau, IIB-Sant Pau, Barcelona, Spain; ^4^ Department of Anatomy and Cell Biology, Faculty of Medicine, University of Extremadura, Badajoz, Spain

**Keywords:** atrial branch occlusion, atrial myocardial infarction, ECG, endocardial electrical mapping, multifrequency myocardial impedance, anatomopathology

## Abstract

**Background:** Characterization of atrial myocardial infarction is hampered by the frequent concurrence of ventricular infarction. Theoretically, atrial infarct scarring could be recognized by multifrequency tissue impedance, like in ventricular infarction, but this remains to be proven.

**Objective:** This study aimed at developing a model of atrial infarction to assess the potential of multifrequency impedance to recognize areas of atrial infarct scar. Methods: Seven anesthetized pigs were submitted to transcatheter occlusion of atrial coronary branches arising from the left coronary circumflex artery. Six weeks later the animals were anesthetized and underwent atrial voltage mapping and multifrequency impedance recordings. The hearts were thereafter extracted for anatomopathological study. Two additional pigs not submitted to atrial branch occlusion were used as controls.

**Results:** Selective occlusion of the atrial branches induced areas of healed infarction in the left atrium in 6 of the 7 cases. Endocardial mapping of the left atrium showed reduced multi-frequency impedance (Phase angle at 307 kHz: from −17.1° ± 5.0° to −8.9° ± 2.6°, *p* < .01) and low-voltage of bipolar electrograms (.2 ± 0.1 mV vs. 1.9 ± 1.5 mV vs., *p* < .01) in areas affected by the infarction. Data variability of the impedance phase angle was lower than that of bipolar voltage (coefficient of variability of phase angle at307 kHz vs. bipolar voltage: .30 vs. .77). Histological analysis excluded the presence of ventricular infarction.

**Conclusion:** Selective occlusion of atrial coronary branches permits to set up a model of selective atrial infarction. Atrial multifrequency impedance mapping allowed recognition of atrial infarct scarring with lesser data variability than local bipolar voltage mapping. Our model may have potential applicability on the study of atrial arrhythmia mechanisms.

## 1 Introduction

Improving electrophysiological identification of areas of atrial fibrosis would be of relevance in the ablation treatment of patients suffering from atrial arrhythmias. Nowadays the electroanatomic cardiac navigators use the information of the local voltage as surrogate marker of fibrosis. However, the voltage cut-off values vary from patient to patient and are dependent on the cardiac rhythm at the time of the procedure. (Lahuerta et al., 2022). Electrophysiological characterization of atrial infarct scarring is hampered by the paucity of animal models mimicking this clinical entity. To date, the basic knowledge on the intrinsic electrophysiological alterations induced by isolated atrial myocardial infarction (MI) are based on short series of experimental studies. (Sinno et al., 2003; Rivard et al., 2007; Nishida et al., 2011; Alasady et al., 2013; Avula et al., 2018; Amorós-Figueras et al., 2020) These studies showed slowing of local atrial conduction, prolongation of the refractory period, (Sinno et al., 2003), and changes in ST segment and voltage of local atrial electrograms. (Amorós-Figueras et al., 2020) More recently new surrogates for the detection of atrial fibrosis have been proposed and these include strain imaging during left atrial reservoir phase, (Laish-Farkash et al., 2021), or atrial magnetic resonance imaging using late gadolinium enhancement LGE-MRI. (Hopman et al., 2022) Endocardial measurement of myocardial electrical impedance allowed recognition of chronic infarction of the ventricle, (Amoros-Figueras et al., 2018), but its ability to detect the infarct scarring in the atria has not been reported.

This study aimed at developing a closed-chest animal model of selective occlusion of atrial coronary branches to detect atrial infarct scarring by endocardial mapping of multifrequency tissue electrical impedance.

## 2 Materials and methods

### 2.1 Study population

This study involved 9 domestic swine (Landrace-Large White cross). Seven pigs underwent atrial coronary branch occlusion whereas the remaining two were used as controls for endocardial voltage mapping and multifrequency impedance data. The study protocol was approved by the Animal Care and Use Committee of our institution, and fully conformed to the Guide for the Care and Use of Laboratory Animals, eighth ed. (National Research Council. Washington, DC: The National Academies Press, 2010).

### 2.2 Experimental procedures

#### 2.2.1 Induction of chronic atrial myocardial infarction

**FIGURE 1:**
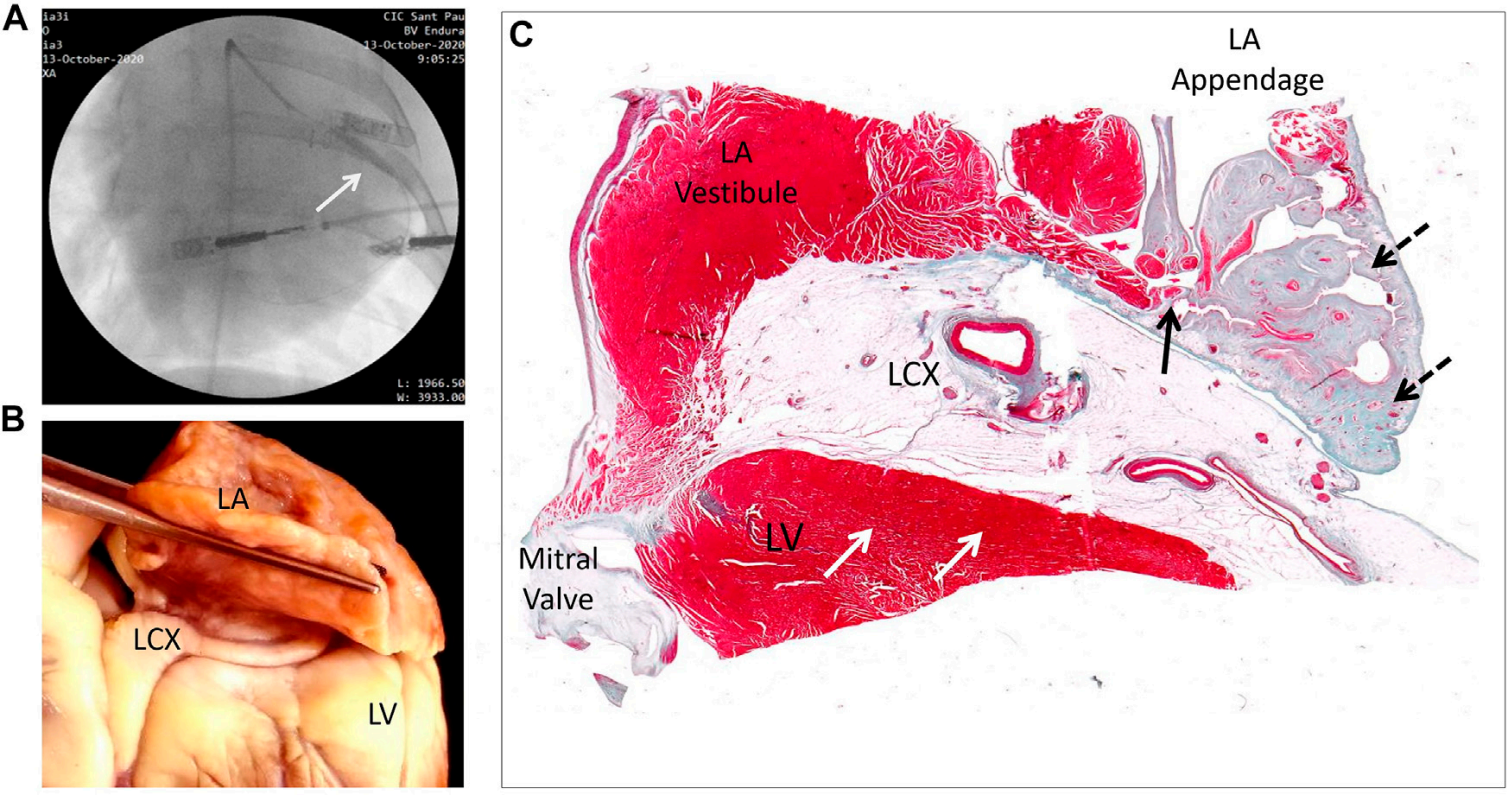
Illustration of the experimental procedure and study specimen preparations. **(A)** Fluoroscopic image of one studied pig showing the catheter balloon located in the proximal segment of the LCX while deploying the covered stent (white arrow). **(B)** Photograph showing the anatomical relationship between the left ventricle (LV), left atrium (LA) and the left circumflex coronary artery (LCX) where the covered stent was deployed. **(C)** Microphotograph of a histological preparation (frontal section) showing both areas of infarct infarcted (dashed arrows) and non-infarcted areas (solid arrows) in the left atrial appendage with preserved left ventricular myocardium (white arrows) corresponding to the annular area.

Seven pigs weighing 49 ± 5 kg were premedicated with midazolam (.6 mg/kg) and ketamine (12 mg/kg) intramuscularly. General anesthesia was induced with intravenous propofol (2–4 mg/kg) and was maintained with a mixture of oxygen and sevoflurane inhalation (2.5%–3.5%). The animals were mechanically ventilated through endotracheal intubation and analgesia was maintained during the entire procedure with intravenous fentanyl (0.1 μg/kg/min). A femoral artery was catheterized with a 7F cannula and a 6F hockey stick guiding catheter (Cordis, United States) was introduced. Under fluoroscopic guidance (Philips Endura), the catheter was advanced to the proximal segment of the LCX and a covered stent (PK Papyrus, Biotronik, Germany) was deployed with a catheter balloon (Figure 1A). We selected this stent because it would allow interruption of blood flow in the exiting atrial coronary branches but preserving at the same time the main stream flow into the LCX, avoiding a concomitant ventricular infarction. We verified the existence of atrial coronary branches arising from the LCX in pigs in a previous series of open-chest experiments. (Amorós-Figueras et al., 2020) The small diameter of these atrial vessels was often below the resolution of our fluoroscopic system and images could not be consistently obtained. A 15-lead ECG was continuously recorded during the procedure to verify the absence of ST-changes secondary to acute ventricular myocardial ischemia. Animals were allowed to recover and were treated with antibiotics and analgesics. After a median period of 6 weeks, the 7 pigs underwent sedation and general anesthesia like in the previous intervention. We performed an atrial endocardial mapping of local electrograms and local tissue electrical impedance. Thereafter, the animals were euthanized, and the hearts processed for anatomopathological study (Figure 1B).

#### 2.2.2 Mapping of left atrial electrograms and tissue electrical impedance

These procedures were performed in the 7 pigs with 6-week old atrial coronary branch occlusion and in the 2 control pigs not submitted to coronary intervention. A femoral vein was catheterized and a mapping electrocatheter (Smarttouch^®^, Biosense-Webster, United States) was advanced into the left atrium through a transeptal access. The electrocatheter was connected to a CARTO three system (Biosense-Webster, United States) to generate 3D high density endocardial mapping of left atrial unipolar and bipolar local electrograms (Electroanatomic map settings: Tissue Proximity Index was activated, Local Activation Time stability was set to 3 ms, Position Stability was set to 2 mm, the map density for voltage maps was 1,646 ± 1,277 points and for impedance maps was 28 ± 9 points, Force Setting was set to Force above minimum threshold, Color Fill Threshold was set to 30). The voltage amplitude (mV) was automatically measured in all endocardial signals and thereafter, voltage and activation maps were constructed. Based on the clinically accepted definition of areas of low voltage in endocardial mapping procedures, (Kottkamp et al., 2017), we considered areas of low atrial voltage those with local bipolar voltage <.5 mV.

Myocardial electrical impedance of the left atrium was measured at selected anatomical regions (atrial appendage, septum, anterior and posterior wall) at frequencies ranging from 1 to 1,000 kHz using the same endocardial mapping electrocatheter. This catheter was connected to an impedance recording system made by our group. (Sanchez et al., 2013) Alternating currents (1 ms duration, 1 mA total peak amplitude) of twenty-six frequencies ranging from 1 to 1,000 kHz were injected between the distal electrocatheter pole and a skin reference electrode (Dispersive pad, 3M) placed on the anterior thoracic region. The resultant changes in current voltage were measured between the distal electrocatheter pole and a second thoracic skin reference electrode (ECG pad, 3M). The local impedance was measured at a sampling rate of 60 Hz during the entire duration of the cardiac cycle and stored at 2 s frames. The impedance magnitude (Z) and the phase angle (PA) were measured at all current frequencies. The Z quantifies the drop of voltage amplitude for a given applied current and the PA reflects the time delay between the voltage and current waves which is influenced by structural characteristics of the myocardial tissue. In an attempt to discriminate between healthy and infarcted atrial regions we selected a phase angle cutoff of −12° at 307 kHz based on a previous study in a pig model of chronic left ventricular infarction. (Amorós-figueras et al., 2017)

To support the assumption that atrial fibrosis accounted for the areas of low-voltage and depressed electrical impedance, we analyzed the left atrial voltage mapping and multifrequency impedance data recorded in two control pigs not submitted to atrial branch occlusion. All voltage and impedance recordings were performed with a stable contact tip-catheter force between 10 and 20 g.

#### 2.2.3 Anatomopathology

The explanted hearts of the 7 pigs with atrial coronary branch occlusion were cleaned from blood and fixed in buffered formalin. The atria were separated at the vestibule level of the ventricles and embedded in a paraffin block. Sections (12 µm) of the entire left atrium were mounted and serially sectioned frontally in 12 µm slices, and then stained with Masson’s trichrome (Figure 1C). Morphometric study was performed to evaluate the average extent of atrial fibrosis. In brief, digital images were taken of the sections stained with Masson’s and the areas of fibrosis were delimitated in the sections of maximum extent of infarcted area using ImageJ software. (Schneider et al., 2012) The areas of fibrosis were expressed as a percentage of the entire limits of the sectioned atria. The identification of the areas of fibrosis was done by two expert anatomopathologists under direct visualization of the histologic preparation at proper magnification. We also calculated the mapping-derived voltage and impedance scar-areas (excluding pulmonary veins) using the software of the electroanatomic mapping system.

### 2.3 Statistical analysis

Quantitative data were expressed as the mean ± standard deviation (SD). The degree of variation of the bipolar voltage and impedance magnitude values was assessed by the coefficient of variation (ratio of the standard deviation and its corresponding mean value). The ordinary two-way ANOVA test with Dunnet’s multiple comparison correction was used to assess the statistical significance of changes in bipolar voltages, impedance magnitude and phase angle at different current frequencies, and ST-segment displacement. A *p*-value <.05 was considered significant. All analyses were performed using SPSS v.22.0 software (IBM-SPSS, United States).

## 3 Results

Nine pigs were included in the study. Occlusion of the LCX atrial branches was performed in 7 cases. Among the latter, 6 completed the entire protocol and one pig was lost during anesthesia induction in the 6-week second intervention, though its heart was included in the anatomopathological study. Two pigs non-submitted to atrial branch occlusion were used for the endocardial mapping studies.

### 3.1 Anatomopathological findings

We observed areas of necrosis with collagen deposition in the left atrium in 6 out of the 7 pigs (86%) submitted to atrial branch occlusion. Histological analysis ruled out the presence of areas of ventricular myocardial infarction (Figure 1). In addition, at the time of stent implantation, close monitoring of the 15 lead ECG in all the animals did not show ST segment changes secondary to acute ventricular myocardial ischemia (lead II: .00 ± .02 mV at baseline vs. .00 ± .03 mV after 30 min vs. −.02 ± .02 mV after 6 weeks, both *p* = ns). Figure 1C is a microphotograph of a pig showing areas of fibrosis affecting the left atrial appendage while preserving left ventricular myocardium. The morphometric analysis of the sections showing the greatest infarct extent in the 6 pigs revealed a mean area of fibrosis of about 38% (range 17%–66%) of the entire microscopic field.

### 3.2 Voltage and impedance derived scar areas of the left atrium

**FIGURE 2:**
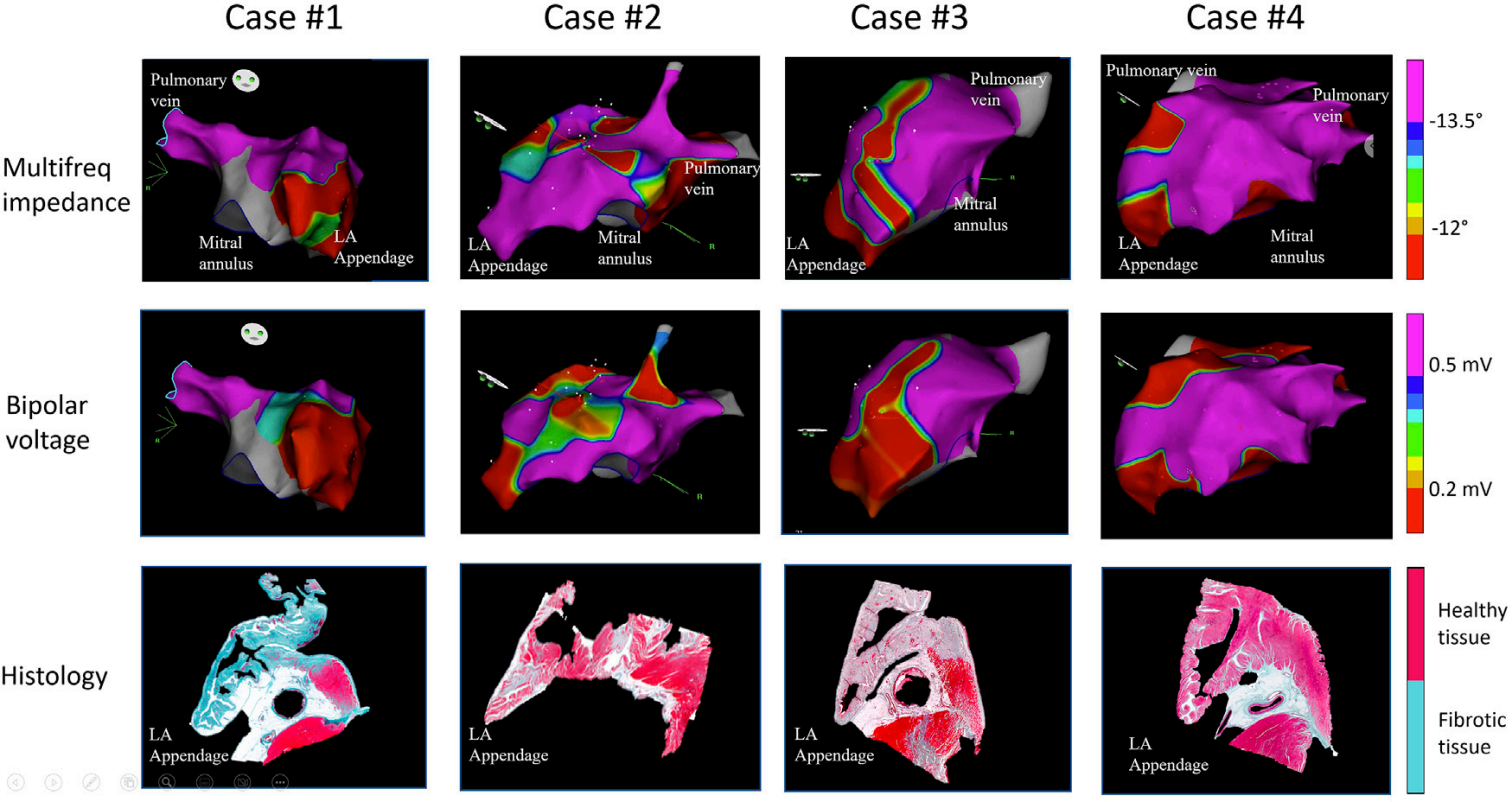
Electroanatomical voltage and multifrequency impedance maps of the left atrium and histological preparations of the explanted hearts of four representative pigs submitted to acute transcatheter occlusion of the atrial coronary artery branches arising from the proximal segment of the left circumflex coronary artery, with preserved left ventricular myocardium. The figure shows a correspondence between endocardial areas of low voltage and impedance and location of infarct zones.

Figure 2 illustrates representative electroanatomical maps of four pigs submitted to atrial coronary branch occlusion. These show areas of low-multifrequency impedance and low-voltage bipolar electrograms in correspondence with atrial anatomical regions affected by the infarction, as observed in the histology sections. The impedance derived scar areas were significantly smaller than the voltage derived scar areas (impedance vs. voltage derived scar area: 31 ± 12 cm^2^ vs. 50 ± 26 cm^2^; *p* < .05). Further electroanatomical mapping analysis of the peri-infarct areas by lowering the voltage scar definition revealed that voltage derived scar areas with a voltage <.1 mV improved the overlap with impedance derived scar areas with a phase angle at 307 kHz lower than −12°. The remaining non-infarcted atrial zones depicted greater voltage and impedance values, but these were lower than those observed in two control pigs not submitted to atrial branch occlusion (Table 1).

**TABLE 1:** Voltage of local left atrial bipolar electrograms in pigs with and without atrial coronary artery branch occlusion.

	Atrial areas with a bipolar voltage cutoff <.5 mV	Atrial areas with a bipolar voltage cutoff ≥.5 mV
Pigs with occluded atrial branches	.2 ± .1 mV (N = 66)	1.9 ± 1.5 mV () (N = 75)
Pigs with non-occluded atrial branches	Not found	3.2 ± 2.2 mV (N = 16)

**, ANOVA *p* < .01 between areas with a bipolar voltage cutoff lower or higher than .5 mV.

### 3.3 Myocardial electrical impedance of the left atrium

**FIGURE 3:**
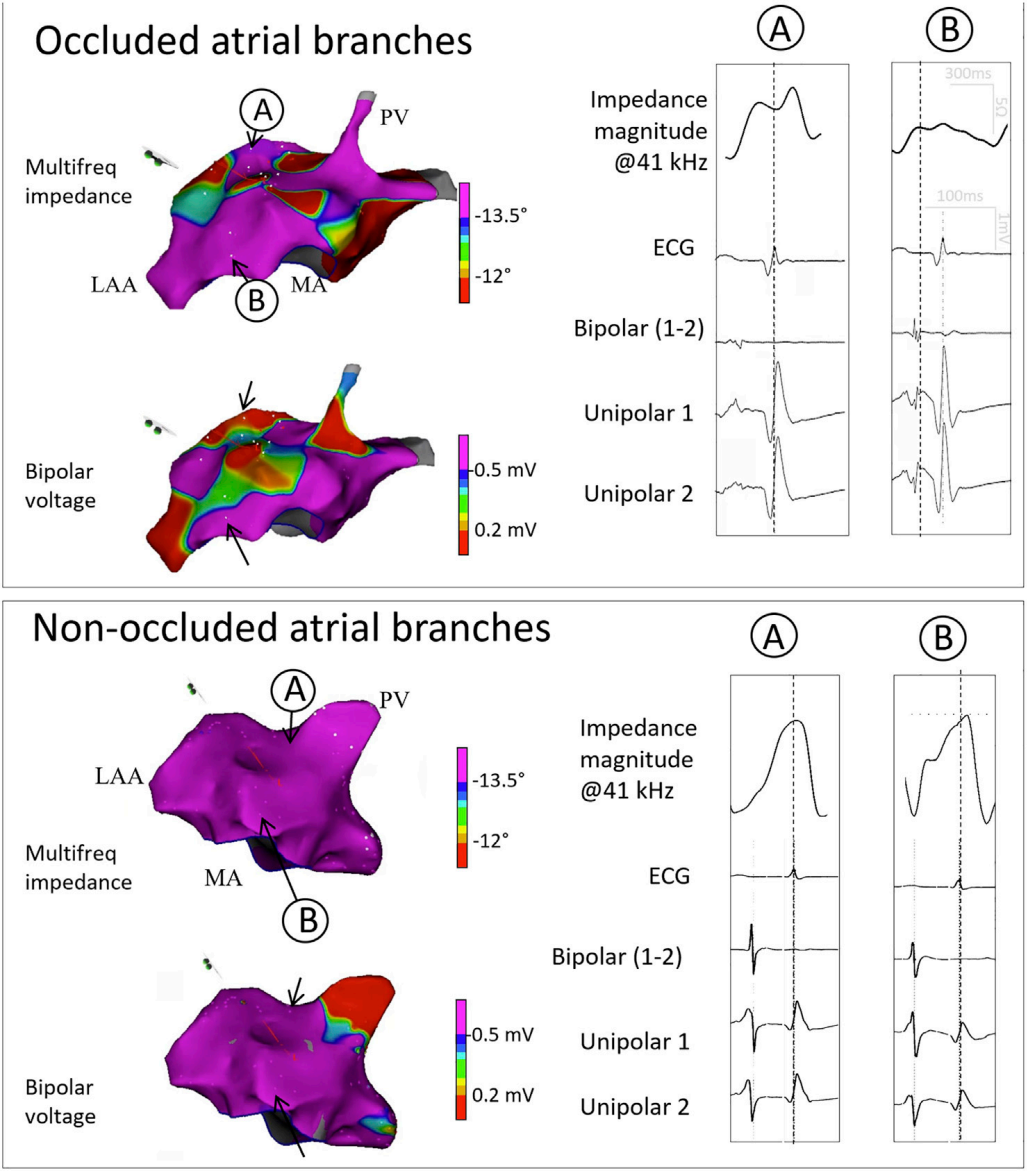
Electroanatomical voltage and multifequency maps and corresponding signals in two representative pigs. The upper panel shows the electroanatomical maps and the corresponding multifrequency impedance, ECG, local bipolar and unipolar signals in two selected sites **(A,B)** in a pig with acute transcatheter occlusion of the atrial coronary artery branches arising from the proximal segment of the left circumflex coronary artery. The lower panel shows the electroanatomical maps and the corresponding signals in two selected sites **(A,B)** in a control pig non-submitted to atrial coronary branch occlusion. LAA: Left atrial appendage; MA: Mitral annulus; PV: Pulmonary vein.

**FIGURE 4:**
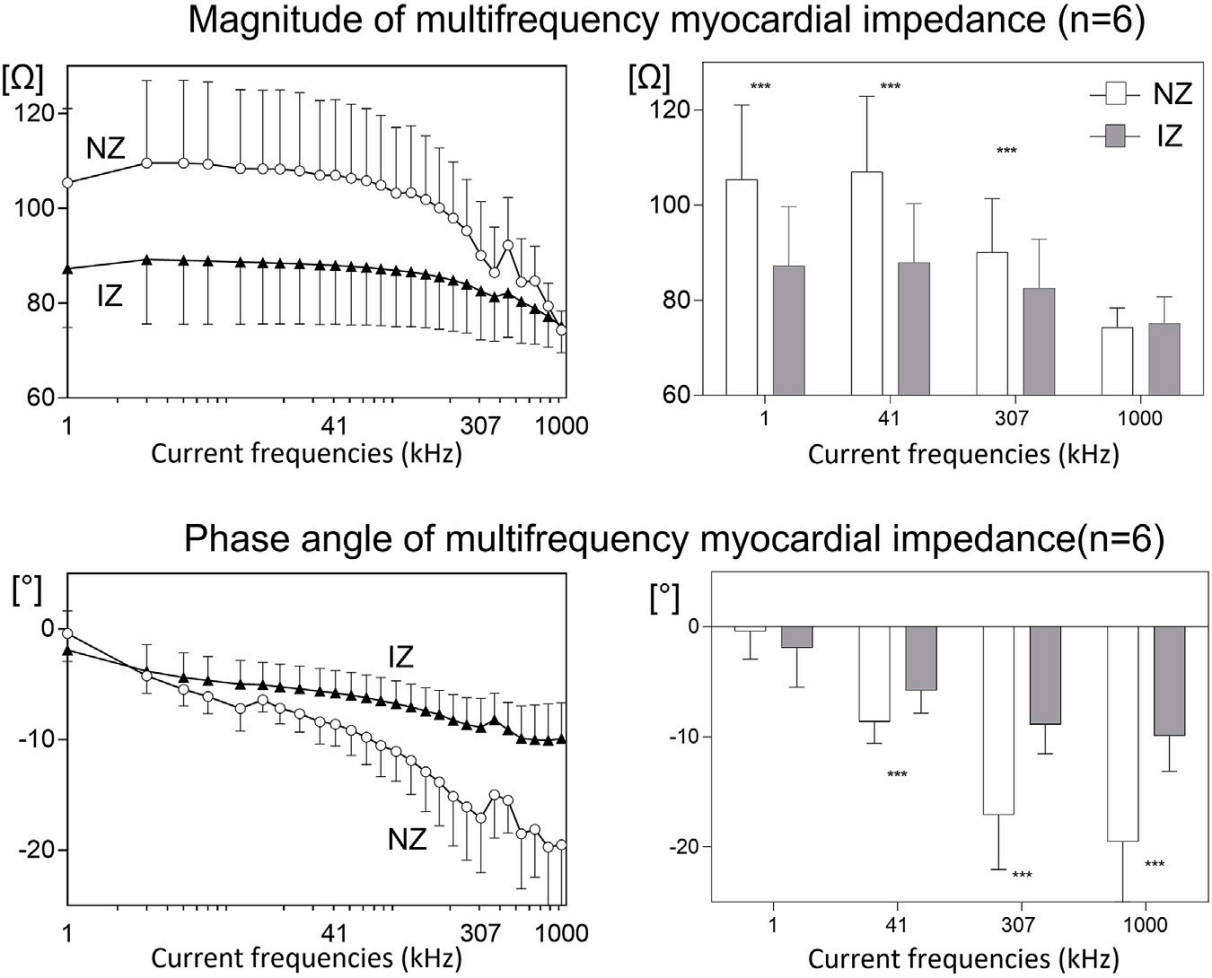
Mean values of the impedance magnitude and phase angle at multifrequency in 6 pigs submitted to acute transcatheter occlusion of the atrial coronary artery branches arising from the proximal segment of the left circumflex coronary artery. The left panels show the mean changes in atrial myocardial impedance magnitude (upper) and phase angle (lower at 26 studied current frequencies. The right panels show the mean changes in atrial myocardial impedance magnitude (upper) and phase angle (lower) at four selected current frequencies. NZ: normal zone; IZ: infarcted zone; ***: ANOVA *p* < .001 between myocardial impedance NZ and IZ areas.

**FIGURE 5:**
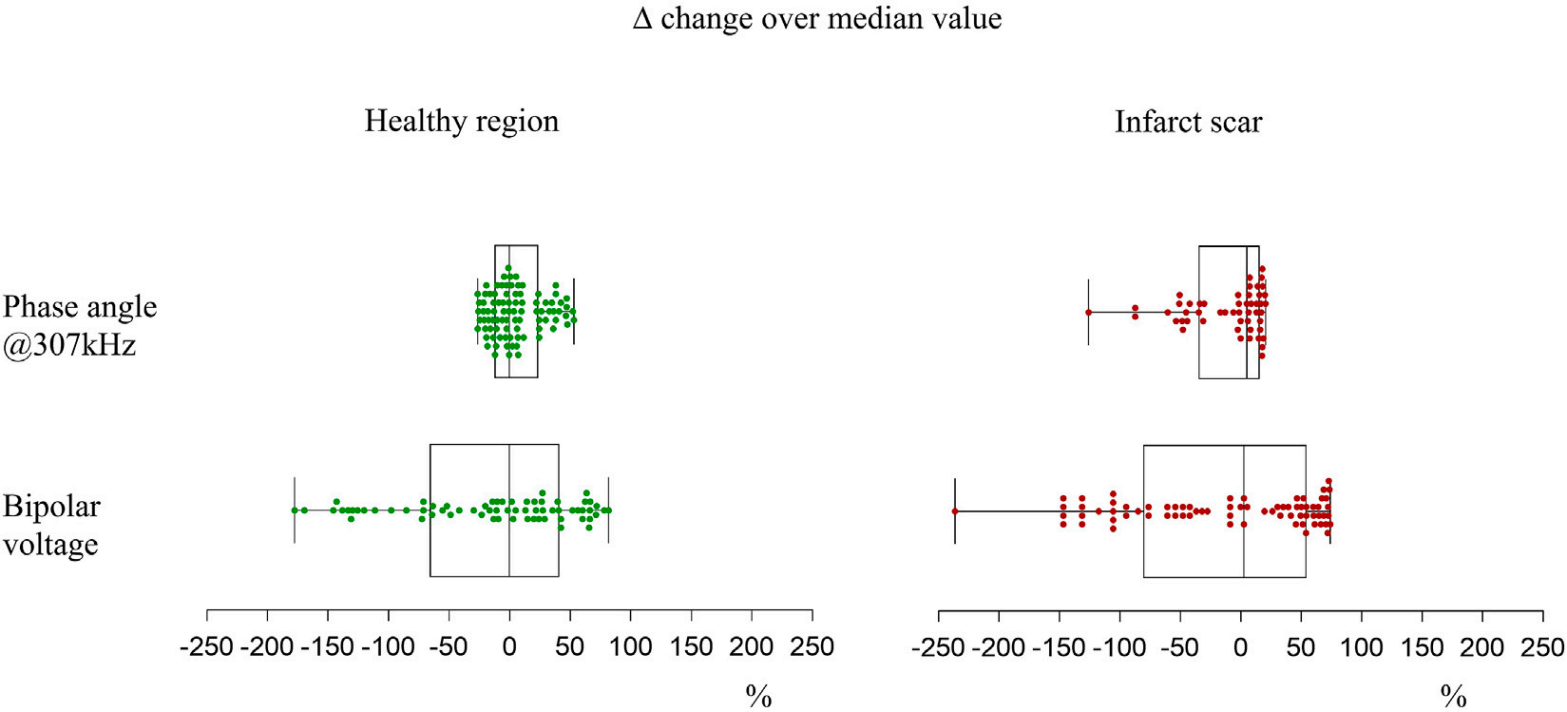
Variability of median values of phase angle of atrial tissue electrical impedance at 307 kHz and bipolar voltage of local atrial electrograms. Data are represented in box and whisker plots. Boxes represent 25th to 75th quartiles; horizontal lines within boxes represent the median; whiskers represent the minimum and maximum values.

Figure 3 shows the bipolar and multifrequency impedance signals in comparable anatomical regions of the left atrium in a pig with occluded atrial coronary branches and in a control pig with non-occluded atrial coronary branches. As shown in Figure 4, the atrial zones affected by the infarction (IZ) showed lower values of the impedance magnitude than non-affected regions (NZ) at all current frequencies. Likewise, the IZ zones showed less negative values of the phase angle at all current frequencies (Table 2). The current frequency range that better discriminated affected and non-affected infarct regions was 1–253 kHz for the impedance magnitude and 200–1,000 kHz for the impedance phase angle. The coefficient of variation of the impedance magnitude was lower than the coefficient of variation of the bipolar voltage in both affected and non-affected infarction regions (coefficient of variation for impedance magnitude at 1,000 kHz vs. bipolar voltage: .30 vs. .77). This can be also observed in Figure 5, where the variability of median values in phase angle of atrial tissue electrical impedance at 307 kHz was lower than the variability of the median values of bipolar voltage.

**TABLE 2:** Magnitude of the atrial myocardial impedance at 1 kHz in pigs with and without atrial coronary artery branch occlusion.

	Atrial areas with a phase angle cutoff < −12°	Atrial areas with a phase angle cutoff ≥ −12°
Pigs with occluded atrial branches	87.3 ± 12.4 *Ω* (N = 53)	105.4 ± 15.6 *Ω* (***) (N = 88)
Pigs with non-occluded atrial branches	Not found	119.7 ± 5.5 *Ω* (N = 16)

**, ANOVA *p* < .001 between areas with a phase angle cutoff lower or higher than −12°.

## 4 Discussion

### 4.1 Main findings

This study shows that myocardial infarction restricted to the atrial chambers can be successfully induced by selective transcatheter occlusion of atrial coronary branches in swine. In this closed-chest model, healed atrial MI was associated with decreased magnitude and phase angle of myocardial impedance at all studied frequencies. Impedance data variability was lower than bipolar voltage values.

### 4.2 Local electrophysiological findings

Left atrial electroanatomical mapping evidenced areas of low voltage of the local electrograms and decreased multifrequency tissue impedance. Post infarct fibrosis is the most likely substrate for these electrophysiologic alterations since left atrial mapping in 2 control pigs not submitted to atrial branch occlusion evidenced normal voltage of electrograms and impedance in similar anatomical atrial regions. Moreover, the infarcted areas observed in the histological preparations shared a correspondence with the anatomical atrial regions showing low voltage electrograms and reduced impedance. A delayed and heterogeneous local activation inside the ischemic atrial myocardium has been reported in dogs after acute or 8-days old occlusion of atrial coronary branches arising from the RCA, (Sinno et al., 2003; Nishida et al., 2011), in sheep after acute occlusion of the LCX, (Alasady et al., 2013), and in pigs with submitted to surgical clamping of atrial branches arising from the LCX. (Amorós-Figueras et al., 2020)

The present study addressed the healing phase of atrial infarct and revealed, for the first time, that the low voltage areas also presented low tissue electrical impedance at all current frequencies. Comparable low-voltage and low-impedance findings were found in pigs with 1-month old ventricular myocardial infarction induced by occlusion of the LAD coronary artery (Amoros-Figueras et al., 2018) thus revealing that both, healed atrial and ventricular myocardial infarction share a similar electrophysiologic substrate. We also found that impedance phase angle values presented less variability than bipolar voltage values, for both healthy and infarct scar regions. This finding, as well as the observation that impedance derived scar areas were significantly smaller than classic voltage derived scar areas (<.5 mV), suggest a more accurate assessment of atrial infarct scar by bioimpedance measurement which, in contrast to voltage mapping, are not dependent on the direction the atrial activation front. (Amorós-figueras et al., 2017) The arrhythmogenic potential of this substrate has been consistently documented in clinical and experimental ventricular myocardial infarction. (Guandalini et al., 2019) However, spontaneous atrial ectopic arrhythmias rarely occurred in experimental atrial infarction, though susceptibility to electrical induction and of atrial fibrillation (AF) has been reported. (Sinno et al., 2003) In our study, vulnerability to electrically induced atrial arrhythmias was not tested.

### 4.3 Anatomopathology

The characteristic structural features of healed atrial infarction in our model were the irregular distribution of the necrotic areas and the sharp delineation of the infarcted border zone. The left atrial appendage was more frequently affected, and the ventricular myocardium was preserved, indicating that deployment of a covered Papyrus stent in the proximal segment of the LCX was a successful method to create a model of isolated atrial myocardial ischemia.

### 4.4 Study limitations

The mean values of the bipolar electrograms and myocardial impedance in atrial areas not affected by the infarction in pigs with occluded coronary branches were lower than those recorded in the healthy atria in pigs with non-occluded coronary atrial branches. This could reflect an electrotonic effect of the infarcted tissue over the neighboring normal zones, or the existence of a non-transmural distribution of the infarct fibrosis over the endocardial non-affected zones.

Due to the individual anatomical variability in the distribution of atrial coronary branches and to their rich inter-anastomotic network, the atrial infarct resulting from the deployment of the covered stent in the proximal segment of the LCX coronary artery was of variable size and location. However, the infarcted areas could be recognized on-line by local mapping of electrograms and local tissue impedance measurements. Spontaneous atrial arrhythmias are seldom in models of atrial infarction, though arrhythmia vulnerability to electrical stimulation can be successfully tested in these models. (Sinno et al., 2003; Nishida et al., 2011; Alasady et al., 2013). Moreover, an infarct healing period longer than that followed in our study (6 weeks) might be a more realistic substrate for spontaneous arrhythmia.

A close point-by-point merging analysis between the infarcted areas in the histological preparations and the regions of low-voltage in the endocardial mapping is challenged by the unpredictable magnitude of the changes in size and volume of the atria secondary to heart explantation and formaldehyde inclusion. A strict site-to-site correspondence of low-voltage electrograms and infarcted areas was not therefore performed in our model. However, we afforded evidence that regions with the low-voltage and low multifrequency impedance mainly corresponded with the areas with atrial infarct involvement.

### 4.5 Clinical implications

In the present work we have mapped the endocardial regions of healthy and infarcted left atrium and found that the areas with low voltage of electrograms depict greater range of variability than the multifrequency impedance values in the same areas. A likely explanation is that local multifrequency measurements explore the stable passive properties of the tissue while the endocardial voltage is affected by dynamic changes in the local activation front. Considering the similarities between human and swine cardiac ischemic electrophysiology, (Lelovas et al., 2014), a model of infarct atrial infarction like the developed in our study emerges as a reliable tool for further research on atrial electrophysiological mechanisms and development of atrial ablation techniques. A major goal for the success of electrical ablation of the arrhythmias is the accurate characterization of the target ablation structure. In this regard, recent studies have highlighted the advantages of the local measurement of tissue electrical impedance to achieve a better pulmonary vein isolation in the ablation of atrial arrhythmias. (Das et al., 2021; García-Bolao et al., 2022)

In these studies, only the magnitude component of the impedance was measured. In our study we have measured both, the magnitude and the phase angle components of the impedance at different frequencies. We have previously reported that multifrequency impedance mapping can detect different degrees of myocardial fibrosis (Amorós-figueras et al., 2016) and, remarkably, the impedance measurement will not be influenced by the changes in cardiac activation sequence. (Amorós-figueras et al., 2017)

## Data Availability

The raw data supporting the conclusion of this article will be made available by the authors, without undue reservation.
